# Large Scale Solid-state Synthesis of Catalytically Active Fe_3_O_4_@M (M = Au, Ag and Au-Ag alloy) Core-shell Nanostructures

**DOI:** 10.1038/s41598-019-43116-7

**Published:** 2019-04-29

**Authors:** Srinivasa Rao Nalluri, Ravikiran Nagarjuna, Dinabandhu Patra, Ramakrishnan Ganesan, Gopalan Balaji

**Affiliations:** Department of Chemistry, Birla Institute of Technology and Science (BITS) Pilani, Hyderabad Campus, Jawahar Nagar, Shameerpet Mandal, Hyderabad, 500078 India

**Keywords:** Environmental impact, Sustainability

## Abstract

Solvent-less synthesis of nanostructures is highly significant due to its economical, eco-friendly and industrially viable nature. Here we report a solid state synthetic approach for the fabrication of Fe_3_O_4_@M (where M = Au, Ag and Au-Ag alloy) core-shell nanostructures in nearly quantitative yields that involves a simple physical grinding of a metal precursor over Fe_3_O_4_ core, followed by calcination. The process involves smooth coating of low melting hybrid organic-inorganic precursor over the Fe_3_O_4_ core, which in turn facilitates a continuous shell layer post thermolysis. The obtained core-shell nanostructures are characterized using, XRD, XPS, ED-XRF, FE-SEM and HR-TEM for their phase, chemical state, elemental composition, surface morphology, and shell thickness, respectively. Homogeneous and continuous coating of the metal shell layer over a large area of the sample is ascertained by SAXS and STEM analyses. The synthesized catalysts have been studied for their applicability towards a model catalytic hydrogen generation from NH_3_BH_3_ and NaBH_4_ as hydrogen sources. The catalytic efficacy of the Fe_3_O_4_@Ag and Ag rich alloy shell materials are found to be superior to the corresponding Au counterparts. The saturation magnetization studies reveal the potential of the core-shell nanostructured catalysts to be magnetically recoverable and recyclable.

## Introduction

**Figure 1:**
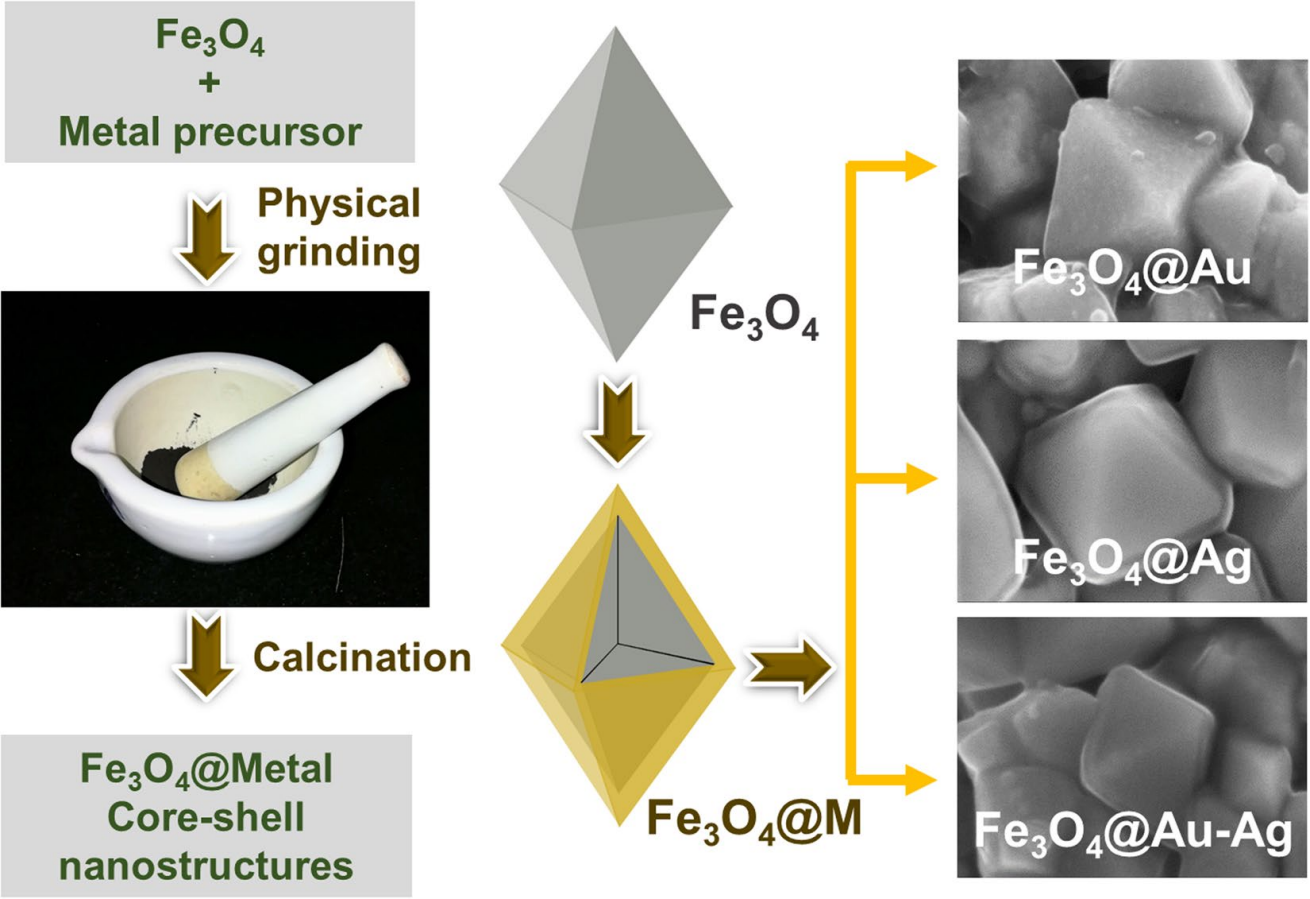
Schematic representation of the solid state synthesis methodology to obtain Fe_3_O_4_@M core-shell nanostructures. The right-hand side images are representative SEM images of the core-shell systems reported in this article.

Core-shell nanostructures are of huge interest to the scientific community due to the enormous potential of these structures towards various applications like catalysis, plasmonic, sensing, energy harvesting, environmental, drug delivery, cell therapy, and cancer treatment^1–9^. Some of the established synthetic routes to obtain core-shell architectures include wet and dry chemical methods such as sonochemical^10^, microwave^11^, hydrothermal^12^, chemical vapor deposition, pulsed-laser-induced dewetting etc^13–17^. Typically, such core-shell nanostructures have been synthesized in a process, wherein a shell layer is deposited over a pre-formed core^18,19^. Particularly, structures like metal oxide@metal (MO@M) are commonly synthesized through solution-based methods that involve coating of metals or metal nanoparticles in the presence of oxide core nanoparticles^18,19^. For example, in the case of Fe_3_O_4_@Au nanoparticles, researchers have utilized epitaxial growth approach, wherein Au^3+^ was reduced in the presence of Fe_3_O_4_ core using various reducing agents such as sodium citrate, sodium borohydride, glucose etc^20^. This method suffers a set back as individual self-nucleated Au nanoparticles may also be formed during the reaction and a separate purification step is needed. In another approach, a small amount of Au nanoparticles were adsorbed onto Fe_3_O_4_ nanoparticles that act as a seed layer for further growth of the metal shell. This approach requires the surface of the core to be chemically modified or pre-conditioned for favorable chemical or electrostatic interactions^20^. Despite the enormous potential offered by these type of core-shell nanostructures, their utility at the industrial level is limited by the solution-phase synthetic approaches that lack scalability, the requirement of further purification step, and the resulting high cost. Furthermore, some of the core-shell nanostructures e.g. metal sulfide@metal selenide, the syntheses are mainly carried out in harmful organic solvents like trioctylphosphine-trioctylphosphine oxide mixture^21^. Solid-state synthesis of metal nanoparticles (Pd and Au) anchored onto support (TiO_2_ and carbon) has been reported by physically mixing the precursor with the support and subsequent calcination^22^. Such systems studied in the literature do not represent core-shell nanostructures^23^. On the other hand, the formation of a continuous metal film over oxide support from a molten metal at high temperatures is a long standing problem, wherein the uniform spreading of the metal film is inhibited by the formation of ridges on the oxide surface^24,25^. Therefore, any factor that decreases the formation of ridges or decreases the surface tension of the molten metal will lead to the formation of a continuous metal film. Here, we demonstrate the fabrication of Fe_3_O_4_@M nanostructures, where M = Au, Ag and Au-Ag alloy, through our solid state synthetic approach. This approach involves simple physical grinding of the metal precursor over commercial Fe_3_O_4_ core followed by calcination (Fig. 1). In this study, we have chosen magnetite as the oxide core candidate, since it offers properties like magnetic recoverability in catalysis, magnetic hyperthermia, magnetoplasmonic etc^26–31^. In case of metal shells, gold has been chosen for its well-known plasmonic^32^, catalytic and biological applications, whilst silver has been chosen due to its dominant plasmonic and anti-microbial properties^33–39^. We hypothesized that coating a noble metal-containing surfactant-based precursor over an oxide core as a thin film followed by metalization would result in a continuous metal shell formation. Therefore, keeping the coatability in mind, different metal precursors such as gold-tetraoctylammonium bromide complex (Au-TOAB) for Au and silver N-lauroyl sarcosinate (Ag-NLS) for Ag have been chosen. Au-TOAB is known for its film formability and therefore expected to yield a good coating over the magnetite core during the synthesis^40^. Since surfactants possessing amphiphilic properties are known to form good quality films, N-lauroyl sarcosinate derivative of silver was synthesized. To the best of our knowledge, this is the first report on a metal oxide@metal core-shell architecture obtained through the solid-state synthetic approach. Our solid-phase synthetic approach to fabricate core-shell nanostructures does not only offer the benefits of cost-effectiveness, near quantitative yield, scalability, and sustainability, but also is an eco-friendly approach where the use of harmful organic solvents is not required. As a bonus, this approach provides additional simplicity, as there is no requirement for any extra separation/purification step.

## Methods

### Materials

Commercial Fe_3_O_4_, HAuCl_4_, tetraoctylammonium bromide, sodium N-lauroyl sarcosinate, and AgNO_3_ were procured from Aldrich. Au-TOAB was synthesized following the literature procedure^40^. Ag-NLS was synthesized by adding a stoichiometric amount of saturated sodium N-lauroyl sarcosinate solution to a solution of AgNO_3_ in a water-ethanol mixture. The obtained white precipitate was washed thoroughly and dried under vacuum for overnight.

### Core-shell synthesis

In a typical core-shell synthesis using our solid-state approach, a calculated amount of the metal precursor was mixed with the magnetite core followed by thorough grinding for 10 min in order to achieve a uniform coating of the precursor over the Fe_3_O_4_ core. The obtained mixture was transferred to an autoclave and subjected to calcination at different temperatures for 2 h. An autoclave was used in order to avoid any surface oxidation during metallization. Typically, the core–precursor assembly after the calcination step was allowed to cool naturally inside the furnace, thus ensuring a slow cooling, unless otherwise mentioned. The resultant products have been labeled as Fe_3_O_4_@M-X, where M and X represent the metal and the weight percent of its corresponding precursor with respect to the initial Fe_3_O_4_ content, respectively.

### Characterization

XRD analysis of the synthesized core-shell nanostructures was performed with Rigaku Ultima IV with Cu K_α_ radiation (λ = 1.5418 Å) at a scan rate of 1°/min. SAXS measurements have been performed using a Rigaku Ultima IV instrument. The measurements have been made from 2θ (${\rm{q}}=\frac{4\pi sin\theta }{{\rm{\lambda }}}$) value of 0.06° to 2° at a scanning speed of 0.03 °/min. The sample was placed and spread over a scotch tape and data were recorded in transmission geometry. For all the samples, the results are presented after due background subtraction. ED-XRF experiments on the core-shell nanostructures were conducted on Panalytical Epsilon-1 instrument. FE-SEM fitted with energy dispersive spectroscopy [Carl-Zeiss ULTRA-55] was utilized to study the surface morphology of the synthesized core-shell nanostructures. HR-TEM images were obtained using JEOL, JEM 2100. The core level XPS were measured using PHI 5000 Versa Prob II (FEI Inc.) to analyze the chemical composition and oxidation states of the constituent metal ions in the core-shell nanostructures. BET surface area measurements on the samples were measured using Micromeritics ASAP 2020 surface area analyzer.

### Catalysis

For hydrogen generation, about 50 mg of the catalyst was added to 18 mL of water taken in a two necked round bottomed flask. One neck of the flask was connected to the gas burette and the other one was sealed with a rubber septum. The solution was thoroughly sonicated for 30 min using bath ultrasonicator to which a solution mixture of 2 mL of 500 mM AB and 250 mM NaBH_4_ in water was introduced using a syringe in order to make the final concentration of AB and NaBH_4_ as 50 and 25 mM, respectively. The pH of the reaction medium was observed to be in the range of 9.2-9.7. The generated hydrogen was collected in the gas burette and the volume of hydrogen was measured as a function of time. For control experiments, either 50 mM of AB or 25 mM of NaBH_4_ solution was separately used, whilst the rest of the parameters were kept identical.

## Results and Discussion

The thermal stability of the precursors has been studied using thermogravimetric and differential thermal analyses. The differential thermal analyses revealed the low melting points of Au and Ag precursors to be 50 and 73 °C, respectively. The thermal decomposition and the corresponding derivative plot of the precursors are given in the Supplementary Information (see Supplementary Fig. S1). The onset of the thermal decomposition in these precursors was found to be close to 200 °C and the major mass loss was found to occur between 230 and 270 °C. Based on the derivative plot, the lowest calcination temperature in this study had been fixed at 250 °C. The calculated and experimental residual mass of the metals in the corresponding precursors is given in Supplementary Table S1.

**Figure 2:**
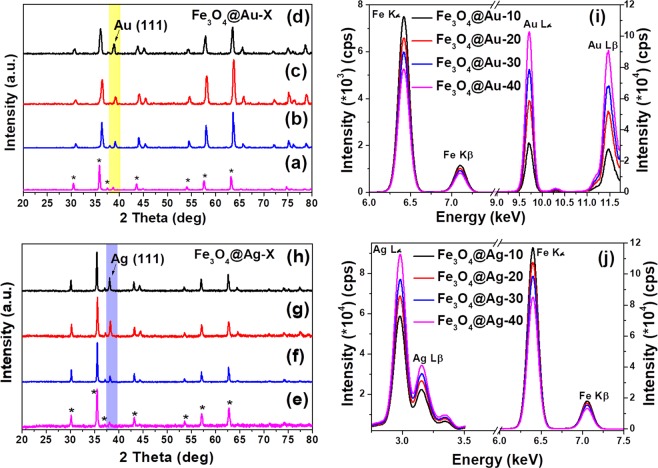
XRD patterns of Fe_3_O_4_@M-X. Top left panel: (**a**) Au-10, (**b**) Au-20, (**c**) Au-30 and (**d**) Au-40; Bottom left panel: (**e**) Ag-10, (**f**) Ag-20, (**g**) Ag-30, and (**h**) Ag-40; ED-XRF spectra of (**i**) Fe_3_O_4_@Au-X (top right panel) and (**j**) Fe_3_O_4_@Ag-X (bottom right panel) at the respective metal edges as indicated in the figure.

The structural and morphological characteristics of commercial Fe_3_O_4_ are given in Supplementary Fig. S3. Figure 2(a–h) shows the powder X-ray diffraction (XRD) patterns of the products obtained from the commercial Fe_3_O_4_ mixed with various loadings of Au-TOAB (a-d) and Ag-NLS (e-h) by calcining at 250 °C for 2 h. It can be observed from the figure that along with the inverse spinel magnetite phase, the characteristic peaks for (111) planes of gold and silver were also observed. As expected, the relative intensity of (111) plane increased with increasing metal precursor loading. This is indicative of the increase in gold and silver content as per the increasing feed ratio.

To further validate and quantify the metal content against the precursor loading, the synthesized core-shell nanostructures were characterized using energy dispersive X-ray fluorescence (ED-XRF). ED-XRF was employed as X-rays in this technique have higher penetration depth (~1-2 µm) and therefore the elemental composition obtained using this technique would be close to the bulk composition. Figure 2(i,j) shows the ED-XRF spectra of Fe_3_O_4_@Au-X (i) and Fe_3_O_4_@Ag-X (j). As seen in Fig. 2(i), the intensity of Fe Kα at 6.4 keV was decreasing with increasing gold content, while the intensity of Au Lα at 9.7 keV was systematically increasing. In the case of Fe_3_O_4_@Ag-X (Fig. 2(j)) also, the Ag Lα at 2.98 keV was systematically increasing with silver loading. The peak intensities fit linearly with increasing the noble metal content (see Supplementary Fig. S4), which confirms that the composition of the resultant core-shell nanostructures is as per the feed ratio.

**Figure 3:**
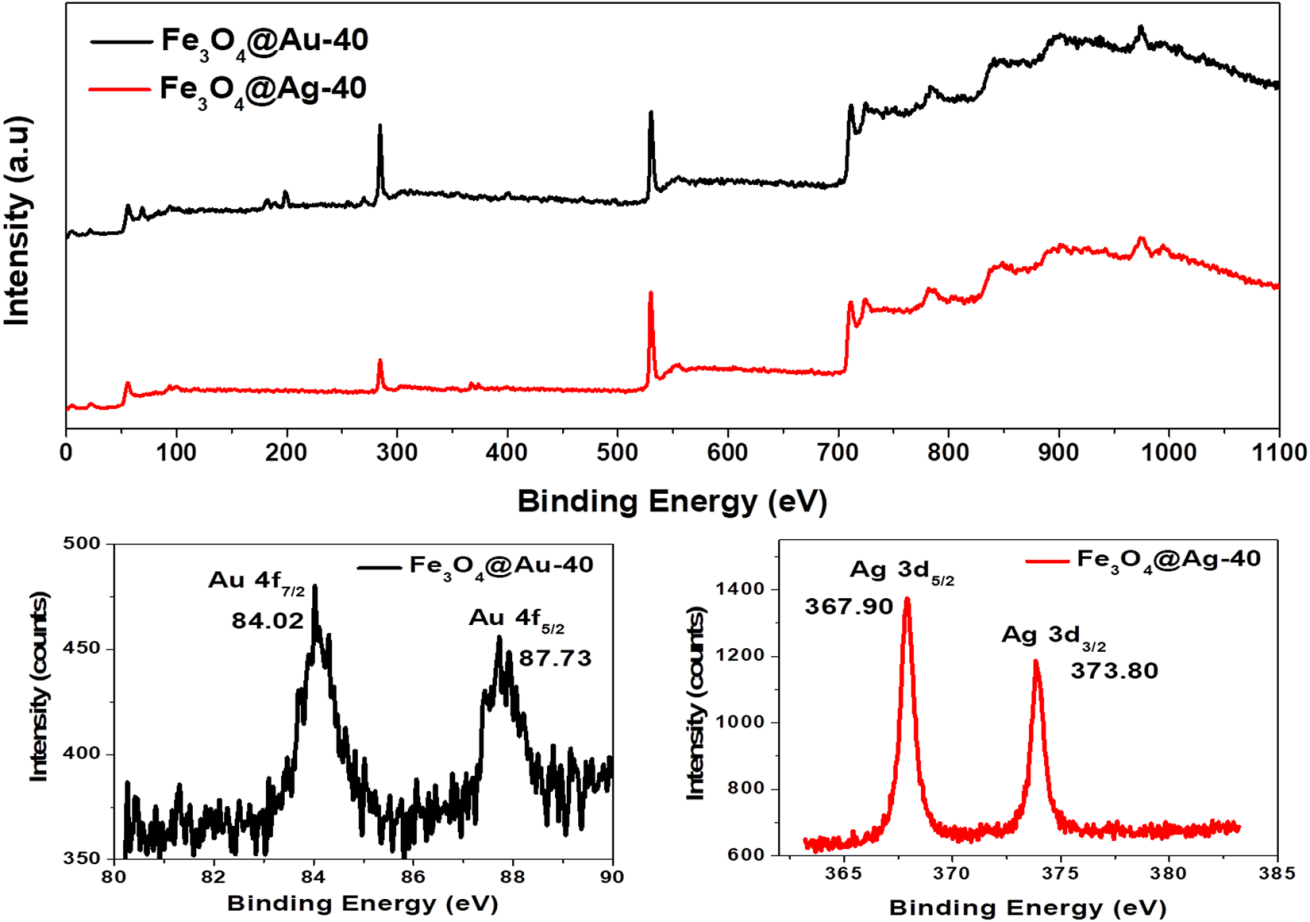
Survey (top panel) and narrow scan (bottom panel) XPS analyses of Fe_3_O_4_@Au-40 and Fe_3_O_4_@Ag40 samples.

X-ray photoelectron spectroscopy (XPS) studies were performed to determine the oxidation state of the metal in the calcined samples, and the results are shown in Fig. 3. The survey scan of the samples showed the presence of the respective metal as well as iron from the magnetite core. The Au 4f core level narrow scan of Fe_3_O_4_@Au-40 revealed two peaks at 84.02 and 87.73 eV corresponding to Au 4f_7/2_ and Au 4f_5/2_ levels, respectively. These binding energy values along with the separation value of 3.7 eV confirmed the oxidation state of gold to be zero. The Ag 3d core level narrow scan of Fe_3_O_4_@Ag-40 showed the characteristic Ag 3d_5/2_ and Ag 3d_3/2_ peaks at 367.6 and 373.52 eV, respectively. In this case, the binding energy values and their separation (~6 eV) confirmed the presence of silver in its (+1) oxidation state as in Ag_2_O. It is known that the surface of silver gets oxidized upon exposure to air. Since XPS is a surface sensitive technique, it reveals the presence of silver oxide in the surface, while XRD ascertained the bulk film (in the shell layer) as metallic silver. Similar features were observed with the XPS spectra corresponding to the lesser loading of the precursor as in Fe_3_O_4_@Au-10 and Fe_3_O_4_@Ag-10 (see Supplementary Fig. S5).

**Figure 4:**
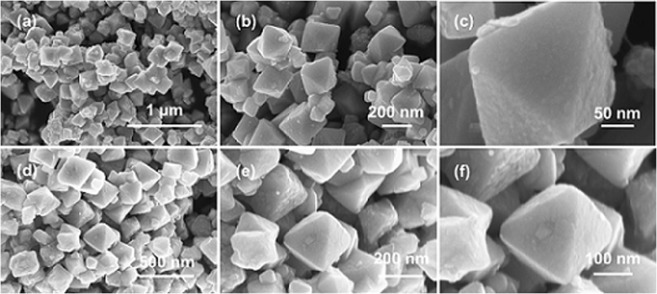
FE-SEM images of Fe_3_O_4_@Au-10 (**a**–**c**) and Fe_3_O_4_@Ag-10 (**d**–**f**) at various magnifications.

Figure 4 shows the field emission scanning electron microscopy (FE-SEM) images of Fe_3_O_4_@Au-10 and Fe_3_O_4_@Ag-10. The commercial Fe_3_O_4_ was found to be a regular octahedron in shape with its base in the range of 150-250 nm and edge in the range of 100-150 nm (data not shown). The FE-SEM analysis of Fe_3_O_4_@Au-10 (Fig. 4(a–c)) clearly revealed the coating of metal over the magnetite core, thus forming a MO@M core-shell nanostructure. In addition to the wavy surface that substantiates the metallic shell formation, a fusion of several magnetite cores mediated by the metal was also observed. Similar MO@M core-shell formation was observed in the case of Fe_3_O_4_@Ag-10 as well (Fig. 4(d–f)). These results confirm the successful fabrication of core-shell nanostructures through the physical grinding approach. The mechanism of the metal shell formation can be envisaged as follows: (i) The surfactant-based precursors possess relatively low melting points that assist in conformal precursor coating over the magnetite core. (ii) The surfactants present in the precursors also help in minimizing the surface energy during the metallization step.

**Figure 5:**
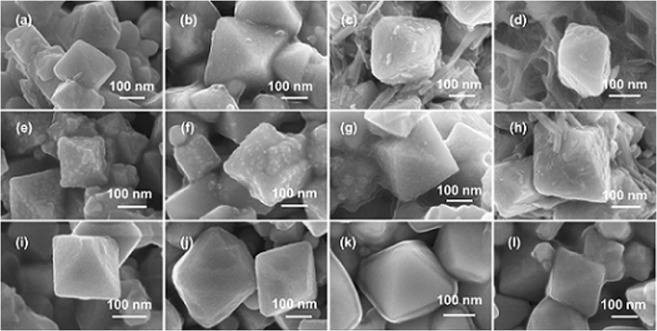
High magnification FE-SEM images of Fe_3_O_4_@Au-X. Top panel: Naturally cooled (**a**) Au-20, (**b**) Au-30, (**c**) Au-40, and (**d**) Au-50; Middle panel: Rapidly cooled (**e**) Au-10, (**f**) Au-20, (**g**) Au-30, and (**h**) Au-40; Bottom panel: Naturally cooled (**i**) Ag-20, (**j**) Ag-30, (**k**) Ag-40, and (**l**) Ag-50.

The surface morphology studies using FE-SEM with varying gold content have been shown in Fig. 5. With increasing loading of the gold precursor a few free metal nanoparticles had also been observed along with the core-shell structures (Fig. 5(a,b)). In addition, more metal-mediated inter-particle conjunction was found with increasing precursor loading. In case of Fe_3_O_4_@Au-40 and Fe_3_O_4_@Au-50 (Fig. 5(c,d)), several Au nanoneedles were found to co-exist along with the core-shell nanostructures, indicating a large excess amount of the metal precursor over the magnetite. This excess amount of precursor that may have been segregated resulting in the nanoneedle formation upon decomposition. In the case of silver systems, the shell was found to be much smoother than in the case of gold. It is noteworthy that the FE-SEM images of Fe_3_O_4_@Ag-40 (Fig. 5(k)), and Fe_3_O_4_@Ag-50 (Fig. 5(l)) revealed a much smoother silver coating over the magnetite core even at this high loading. However, the metal-mediated aggregation of the core-shell nanostructures with an increase in metal precursor loading was evident. It is remarkable that despite having slightly higher metal content (see Supplementary Table S1), silver did not form a significant amount of free metal nanoparticles/nanoneedles as opposed to gold. In order to gain insight to the shell formation mechanism, the core–gold precursor assembly was subjected to a rapid cooling by immersing the autoclave into an ice-cold water set up immediately after the 2 h calcination. In the case of Fe_3_O_4_@Au-10 (Fig. 5(e)), several hump type structures in the shell layer of gold was visible over the shell, indicating that the gold precursor initially formed nanoparticles during the decomposition step, which further melted or diffused to form a continuous thin film^41^. With the increment of gold precursor as in Fe_3_O_4_@Au-20 (Fig. 5(f)), the rapid cooling revealed relatively lesser amount of humps in the shell, but resulted in more metal-mediated particle aggregation, indicating the higher amount of the gold. In case of Fe_3_O_4_@Au-40 (Fig. 5(h)), the rapid cooling showed a small number of nanoneedles along with core-shell structures similar to the slow cooling process that reaffirmed the excess amount of gold precursor as discussed earlier. These observations can be interpreted in terms of surface energy. The surface energies of Au and Ag are 1500 mJ/m^2^ and ~1250 mJ/m^2^, respectively^42^. As Au has higher surface energy, it has an inherent tendency to ball up, leading to segregated nanoparticles at higher loadings. Although Ag also has a similar surface energy, its oxidized form is known to possess surface energy in the range of 30 to 50 mJ/m^2^ ^43^. Therefore, the formation of thin oxide layer (evidenced by XPS) on the very surface of Ag shell could have further assisted in minimizing the surface energy, which could have additionally contributed for a relatively smoother shell surface with less aggregation, when compared to Au. The low magnification FE-SEM images of the samples are presented in the Supplementary Information (see Supplementary Figs S6 and S7).

**Figure 6:**
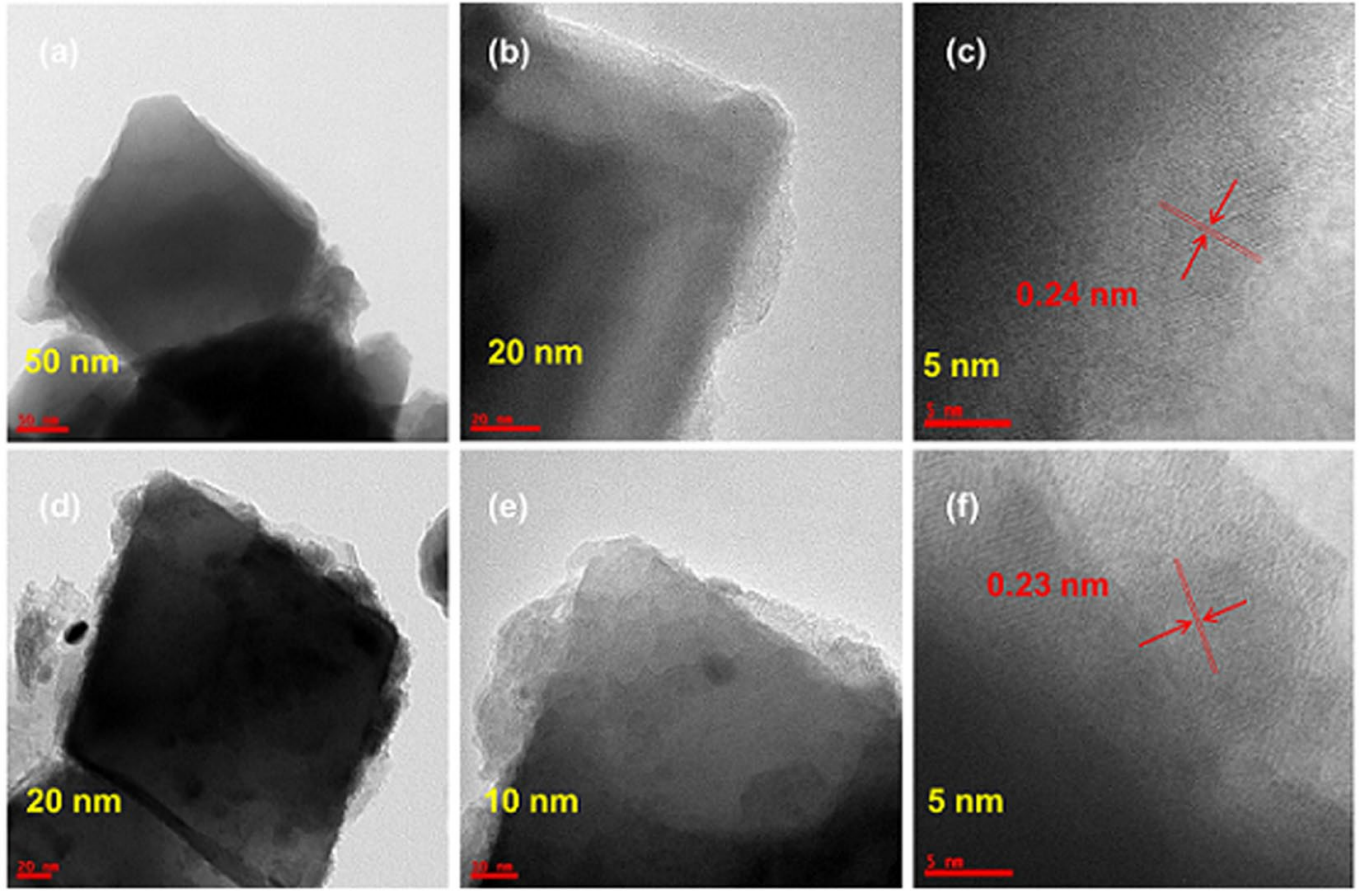
HR-TEM images with various magnifications of Fe_3_O_4_@Au-40 (**a**–**c**) and Fe_3_O_4_@Ag-40 (**d**–**f**). The lattice fringes spacing of 0.24 and 0.23 correspond to the (111) plane of Au and Ag, respectively.

High-resolution transmission electron microscopy (HR-TEM) analysis was performed over selected samples to investigate the surface coverage of the metal shell. The HR-TEM images of Fe_3_O_4_@Au-10 showed complete coverage of Fe_3_O_4_ with Au, confirming the core-shell formation (see Supplementary Fig. S8). The higher magnification image revealed the shell thickness in the range of ~5-9 nm. Similar surface coverage was observed with Ag-shell as in Fe_3_O_4_@Au-10 (see Supplementary Fig. S8). In the case of Fe_3_O_4_@Au-40 and Fe_3_O_4_@Ag-40 (Fig. 6), such core-shell nanostructures were clearly visible. In these cases, the shell thickness was found to be slightly higher and rougher than that in Fe_3_O_4_@Au-10. The respective fringes with lattice spacing of 0.24 and 0.23 nm characteristic of (111) planes of gold and silver corroborated the crystallinity of the metals as confirmed by XRD.

Brunauer–Emmett–Teller (BET) surface area measurements on selected samples were performed to understand the effect of precursor loading in inter-particle aggregation. The surface area of the commercial Fe_3_O_4_ was determined to be 7.5 m^2^/g. Compared to this, Fe_3_O_4_@Au-10 and Fe_3_O_4_@Ag-10 showed a decreased surface area of 4.6 and 4.9 m^2^/g, respectively. When the precursor loading was increased to 40%, the surface area of the corresponding Au and Ag samples further decreased to 2.6 and 3.9 m^2^/g, respectively. While the decrease in surface area with both the metals reveal the occurrence of aggregation, the data also suggest that the aggregation is pronounced to a greater extent in case of Au. This observation is in line with our FE-SEM analyses.

**Figure 7:**
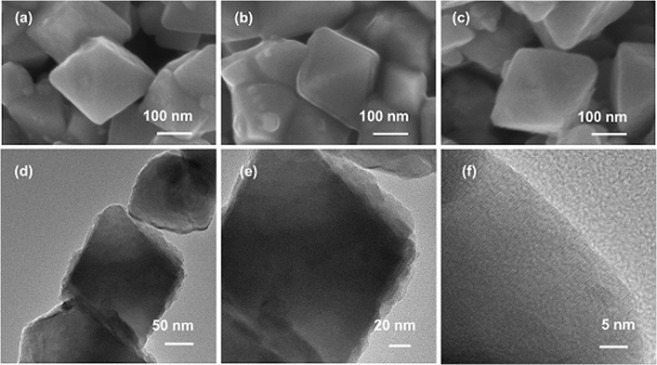
FE-SEM images of (**a**) Fe_3_O_4_@AuAg-15:5, (**b**) Fe_3_O_4_@AuAg-10:10 and (**c**) Fe_3_O_4_@AuAg-5:15 at various magnifications; HR-TEM images (**d**–**f**) with various magnifications of Fe_3_O_4_@AuAg-10:10.

We further continued to explore the potential of fabricating metal oxide@alloy core-shell nanostructures by making Au-Ag alloys of different compositions such as 1:3, 1:1 and 3:1 over the Fe_3_O_4_ core. The total amount of metal precursors was maintained as 20% with respect to Fe_3_O_4_. The XRD results showed that the peak of (111) plane of the Au-Ag alloy was positioned between the 2θ values of pure Au and Ag (see Supplementary Fig. S9). For these alloys, the ED-XRF showed that the Au Lα peak was increasing with gold content in the alloy and simultaneously showing the decrease in the intensity of Ag Lα peak (see Supplementary Fig. S10). It is noteworthy that a comparison with the calibration curve confirmed the homogeneous composition of the alloys as per the metal feed ratio. The FE-SEM analyses have shown smooth coverage (lower surface energy due to silver oxidation) of the AuAg alloy as a shell layer on top of the Fe_3_O_4_ core (Fig. 7(a–c) and see Supplementary Fig. S11), which was corroborated by HR-TEM analysis on Fe_3_O_4_@AuAg-10:10 (Fig. 7(d–f)). Additional scanning tunneling electron microscopy (STEM) analyses of Fe_3_O_4_, Fe_3_O_4_@Au-20, Fe_3_O_4_@Ag-20 and Fe_3_O_4_@AuAg-10:10 revealed and confirmed a smooth and continuous metal shell layer coating over the core (see Supplementary Fig. S12). To ascertain the reproducibility and credibility of this solid-state approach, we synthesized SiO_2_@Au-40, SiO_2_@Ag-40, and SiO_2_@AuAg-10:10. The characterization details such as XRD, ED-XRF, STEM and surface area analyses have been presented in the Supplementary Information (see Supplementary Figs S13 and S14).

**Figure 8:**
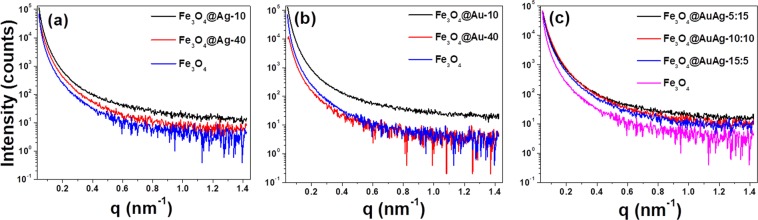
SAXS analyses over of (**a**) Fe_3_O_4_@Ag-X, (**b**) Fe_3_O_4_@Au-X and (**c**) Fe_3_O_4_@AuAg-X alloy core-shell nanostructures.

Small angle X-ray scattering (SAXS) is a powerful technique that reveals the microstructural information of the sample over a large area^44^. In our study, qualitative SAXS analysis studies were performed for selected materials in order to ascertain the structural details in the nanoscale regime (Fig. 8(a–c)). The scattering intensity is dependent on two factors: the constituent elements present and the sample thickness. It is known that the scattering generally increases with increasing atomic number. Pristine Fe_3_O_4_ was used as the control material for discussion. In the case of Ag shell materials, the scattering intensity of Fe_3_O_4_@Ag-10 was more than the pristine Fe_3_O_4_ at all q values. Ag has a higher atomic number and therefore expected to have higher scattering. Thus, the observation indicates that the pristine Fe_3_O_4_ is completely modified on the surface due to the formation of the Ag shell layer. With the increase in Ag content further as in Fe_3_O_4_@Ag-40, the scattering intensity was found to be in between pristine Fe_3_O_4_ and Fe_3_O_4_@Ag-10. And, at high q values, it was difficult to differentiate the scattering between the two Ag samples. The linear absorption coefficient µ is defined as -ln(I_s_/I_0_)t, where I_s_ is the maximum intensity of the sample, I_0_ is the maximum intensity without the sample and t is the thickness of the sample. As the sample was smeared over the scotch tape, any change in I_s_ can be attributed to the change in the µ value. Thus, the observed decrease in the scattering intensity (I_s_) with the increase in Ag loading is due to the increase in µ, which signifies the increasing Ag shell thickness. A similar trend was also observed in Fe_3_O_4_@Au-10, however, for Fe_3_O_4_@Au-40 the scattering intensity was lesser than the pristine Fe_3_O_4_. This can be attributed to the very high µ value due to the thicker Au shell that resulted in a greater loss of scattering intensity. As expected, the scattering intensity of Fe_3_O_4_@Au-10 sample was the highest amongst other samples (see Supplementary Fig. S15). SAXS analyses were also performed on the AuAg alloy shell materials such as Fe_3_O_4_@AuAg-5:15, Fe_3_O_4_@AuAg-10:10 and Fe_3_O_4_@AuAg-15:5. As can be seen from Fig. 8(c), the scattering intensity of alloys is higher than the control Fe_3_O_4_ and similar amongst the compositions at lower q values. However, the scattering intensity, especially at higher q values, gently decreases with increasing gold content in the alloy. These results ascertain the continuous metal shell formation over the Fe_3_O_4_ core.

Although the FE-SEM studies did not reveal much about the uniformity of the shell thickness, the HR-TEM studies have shown that it varies within a core-shell nanoparticle system. This is most likely related to the degree of homogeneity of the initial metal precursor coating before subjecting to calcination. This behavior limited us from exploring the controlled variation of the shell thickness as a function of metal precursor loading. However, SAXS results imply that there is an increase in the average shell thickness over a large area with increasing metal loading.

**Figure 9:**
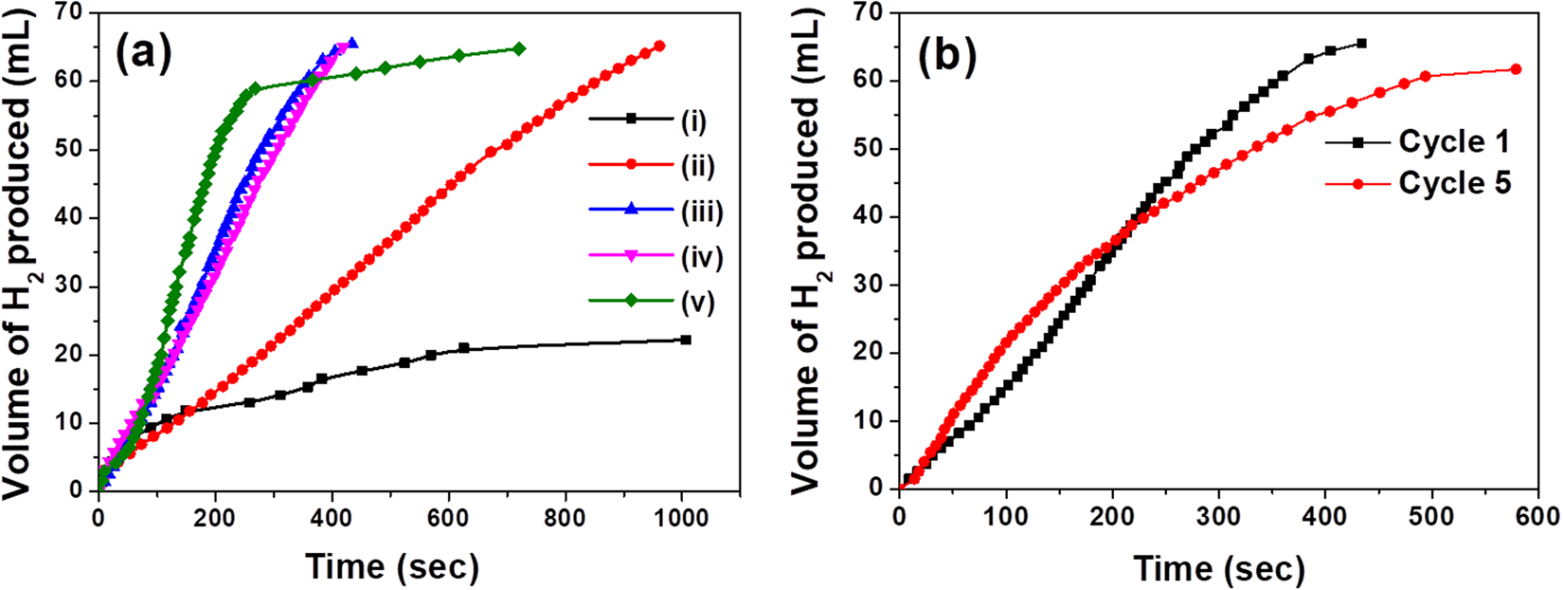
(**a**) Hydrogen generation studies for different catalysts using a mixture of AB and NaBH_4_ as the hydrogen sources: (i) Fe_3_O_4_@Au-20, (ii) Fe_3_O_4_@AuAg-15:5, (iii) Fe_3_O_4_@AuAg-10:10, (iv) Fe_3_O_4_@AuAg-5:15 and (v) Fe_3_O_4_@Ag-20. (**b**) Recyclability studies under same conditions using Fe_3_O_4_@AuAg-10:10. In all cases, [AB] = 50 mM; [NaBH_4_] = 25 mM; Total volume of the solution = 20 mL, Catalyst loading = 50 mg.

The catalytic applicability of the core-shell nanostructures obtained through the solid-state synthetic approach has been studied by choosing catalytic hydrogen generation as a model reaction. Ammonia borane (AB) was chosen as the hydrogen source, as it possesses attractive properties like low molecular weight, easily transportable solid, safe and high gravimetric hydrogen storage^45–47^. The catalytic activity of Fe_3_O_4_@Au-20 and Fe_3_O_4_@Ag-20 catalysts was found to be comparable and in both the cases ~16 mL of hydrogen was generated by ~1150 s (see Supplementary Fig. S16(a)). Hydrogen was also generated using NaBH_4_ as the hydrogen source since it is also considered to be one of the potential hydrogen storage compounds^48,49^. Despite taking half equivalent of NaBH_4_, the total hydrogen generated was higher than the case of only AB. The volume of hydrogen generated was ~30 mL with Fe_3_O_4_@Ag-20 and ~22 mL with Fe_3_O_4_@Au-20 by ~1200 s (see Supplementary Fig. S16(b)). Interestingly, when AB and NaBH_4_ are mixed together in 2:1 molar ratio, the Fe_3_O_4_@Ag-20 was found to be highly active that rapidly produced 60 mL of hydrogen by 270 s. After this time, another 5 mL of hydrogen was slowly produced in an additional 450 s (Fig. 9(a)). With Fe_3_O_4_@AuAg-15:5 alloy, the hydrogen produced was 65 mL by 960 s, which was better than the pure Au catalyst. When the Ag content was increased as in Fe_3_O_4_@AuAg-10:10 and Fe_3_O_4_@AuAg-5:15, the rate of hydrogen generation was found to be high and comparable to that of pure Ag catalyst. With both of these catalysts, ~66 mL of hydrogen (close to 100% of theoretical estimate) was generated by ~430 s. Interestingly, the rate of hydrogen generation with all the alloy catalysts remained almost constant throughout the reaction, which was not the case with pure Ag catalyst that slowed down towards the end of the reaction. The Fe_3_O_4_@AuAg-10:10 catalyst was studied for its kinetics, thermodynamics and magnetic recyclability. Kinetics experiments were performed in order to determine the order with respect to the concentration of catalyst and the hydrogen source mixture. The reaction was found to follow first order kinetics (slope of 0.994) with the catalyst loading in the range of 10 to 50 mg (see Supplementary Fig. S17(a,b)) and the reaction was found to follow zero order kinetics (slope of 0.2) with respect to AB/ NaBH_4_ mixture (see Supplementary Fig. S17(c,d))^50–52^. Similar values for order were obtained for SiO_2_ based catalysts as well, which indicates that the reaction pathway is identical irrespective of the core component (see Supplementary Fig. S18). The thermodynamic studies revealed the activation energy to be 42.4 kJ/mol (see Supplementary Fig. S19(a,b)). The kinetic and thermodynamic parameters have been found to be similar to the works reported in the literature^50–52^. Control experiments were performed, wherein pristine Fe_3_O_4_, SiO_2_, and no-catalyst were employed for the hydrogen generation (see Supplementary Fig. S20). Our results indicate that ~9-12 mL of hydrogen was generated in 600 s in all the three scenarios employed for the reaction. The higher catalytic activity of the core-shell materials indicates the role of the shell layer composition in the hydrogen generation.

The Fe_3_O_4_@AuAg-10:10 catalyst retained similar activity over the successive cycles and the activity was found to remain very similar even after five cycles (Fig. 9(b)). The recycled Fe_3_O_4_@AuAg-10:10 catalysts were characterized using FE-SEM, HR-TEM and ED-XRF (see Supplementary Fig. S21). FE-SEM and HR-TEM revealed that the overall morphology of the core-shell nanostructures remained intact. However, along with the core-shell nanoparticles, some additional mass feature was noticed that could be attributed to the polyborazylene byproduct that might have come along with the catalysts (see Supplementary Fig. S22). It is pertinent to mention that the composition of the recovered catalyst by ED-XRF indicates leaching of silver to the tune of 35.8%, while a minimal leaching of gold to the extent of 8.7% (see Supplementary Fig. S21(d)). The unexpectedly high leaching of silver could be attributed to the possible formation of silver-amine borane complex. Although there have been instances in the literature on the combined use of both AB and NaBH_4_, the exact role of NaBH_4_ in altering the reaction rate is not clearly established^53–57^. It is reported that the release of hydrogen from AB is activated by the presence of bases and borohydrides^58,59^. Hence, in the similar line, our results can be attributed to a synergistic mechanism operating between the two hydrogen sources.

Since the saturation magnetization (M_s_) is an indicator of magnetic recoverability of a catalyst, we performed room temperature vibrating sample magnetometer (VSM) measurements for selected samples and the results are plotted in Supplementary Fig. S23. The saturation magnetization (M_s_) value of the pristine Fe_3_O_4_ sample was reported to be ~174 emu/g^60^. The M_s_ values obtained for Fe_3_O_4_@Au-10, Fe_3_O_4_@AuAg-10:10, Fe_3_O_4_@Ag-10 were 68.5, 82.1 and 98.1 emu/g, respectively. In comparison to the pristine Fe_3_O_4_, the decrease in M_s_ value of Fe_3_O_4_ with the incorporation of Au or Ag can be attributed to the shielding effect of diamagnetic noble metal/alloy shell layer coated over the Fe_3_O_4_ core. The obtained M_s_ values indicate that these materials retain sufficient magnetization, which is crucial for the excellent magnetic recoverability and reusability of the catalysts.

## Conclusions

We have shown a solid-state synthetic approach to fabricate Fe_3_O_4_@M (where M = Au, Ag and Au-Ag alloy) core-shell nanostructures leading to gram scale synthesis in the laboratory conditions. Extensive characterizations using XRD, XPS, ED-XRF, FE-SEM, and HR-TEM analyses have confirmed the successful synthesis of Fe_3_O_4_@M core-shell nanostructures. Furthermore, qualitative SAXS analyses have revealed continuous metal shell formation and thus confirming the core-shell architecture present over a large area of the sample. The Fe_3_O_4_@Ag and Ag-rich alloy catalysts synthesized in this study have shown high catalytic efficacy for hydrogen generation than Fe_3_O_4_@Au and Au-rich alloy. Such a high catalytic efficacy has been found to be profound when a mixture of AB and NaBH_4_ is used rather than employing the hydrogen sources individually. The magnetic recoverability and recyclability studies on Fe_3_O_4_@AuAg-10:10 catalyst has demonstrated the efficient reusability of the catalysts even after 5 cycles. In general, a solventless simple physical grinding of a metal precursor over an oxide core followed by thermolysis is presented as a potential strategy for facile, robust, and cost-effective large scale synthesis of MO@M systems. In addition, the prime merit of this approach is to maintain the metal content in the resulting MO@M systems as per the feed ratio. We believe that this approach will be a paradigm shift for further synthetic explorations on different shell materials such as oxides, sulfides, nitrides, and non-noble metals *via* reduction of an initially formed oxide shell.

## Supplementary information

Large Scale Solid-state Synthesis of Catalytically Active Fe3O4@M (M = Au, Ag and Au-Ag alloy) Core-shell Nanostructures
