# Supplementary material for: Large Scale Solid-state Synthesis of Catalytically Active Fe3O4@M (M = Au, Ag and Au-Ag alloy) Core-shell Nanostructures
Source: Sci Rep. 2019 Apr 29;9:6603. doi: 10.1038/s41598-019-43116-7 (PMC6488626; doi:10.1038/s41598-019-43116-7)
Supplement: Supplementary file 1 — Large Scale Solid-state Synthesis of Catalytically Active Fe3O4@M (M = Au, Ag and Au-Ag alloy) Core-shell Nanostructures [file 41598_2019_43116_MOESM1_ESM.docx]

**Supplementary Information**

Large Scale Solid-state Synthesis of Catalytically Active Fe_3_O_4_@M (M = Au, Ag and Au-Ag alloy) Core-shell Nanostructures

Srinivasa Rao Nalluri,† Ravikiran Nagarjuna,† Dinabandhu Patra, Ramakrishnan Ganesan,* and Gopalan Balaji*

Department of Chemistry, Birla Institute of Technology and Science (BITS) Pilani, Hyderabad Campus, Jawahar Nagar, Shameerpet Mandal, Hyderabad-500078, India.

E-mail: ram.ganesan@hyderabad.bits-pilani.ac.in; gbalaji@hyderabad.bits-pilani.ac.in

**Figure S1.** Thermogravimetric and differential thermal analyses of the metal precursors used in this study. Black, red and blue lines represent thermogravimetry, differential thermogravimetry, and differential thermal analysis curves, respectively.

**Table S1.** Calculated and experimental metal content (wt.%) in the Au and Ag precursors used in this study.

| Sample Code | Calculated | Experimental |
| --- | --- | --- |
| Au-TOAB | 23.2 | 25.4 |
| Ag-NLS | 28.5 | 28.8 |

**Figure S2.** Thermogravimetric analyses of the physically grounded samples of Fe_3_O_4_ core with Au-TOAB precursor before calcination.

**Table S2.** The calculated and experimentally obtained residual mass in different Fe_3_O_4_@Au-X samples.

| **Sample** | **Calculated residual mass (wt.%)** | **Experimental residual mass (wt.%)** |
| --- | --- | --- |
| Fe_3_O_4_@Au-10 | 93.2 | 91.9 |
| Fe_3_O_4_@Au-20 | 87.6 | 86.7 |
| Fe_3_O_4_@Au-30 | 82.3 | 81.3 |
| Fe_3_O_4_@Au-40 | 78.7 | 77.1 |

The close matching between the calculated and experimentally obtained residual mass indicates the homogeneity of the precursor mixing in the physically grounded samples.


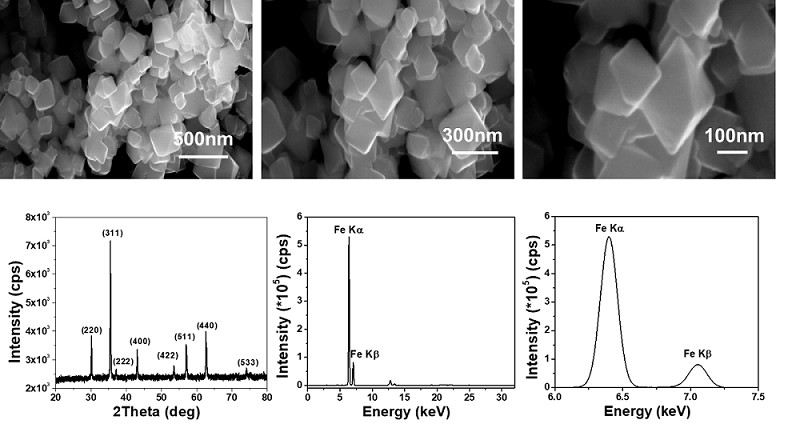


**Figure S3.** Morphology and structural characteristics of commercial Fe_3_O_4_.

**Figure S4.** Calibration curve of the metal content in the Fe_3_O_4_@M-X based on ED-XRF analyses.

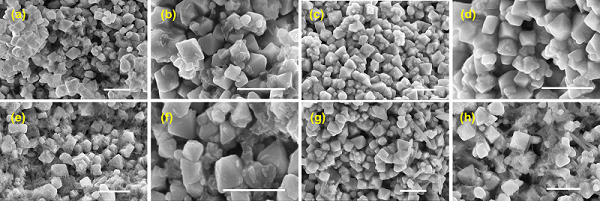
**Figure S5.** XPS survey (top panel) and narrow (bottom panel) scan of Fe_3_O_4_@Au-10 and Fe_3_O_4_@Ag10 samples at the respective edges.

**Figure S6.** Low magnification FE-SEM images of Fe_3_O_4_@Au-X, where X = (a, b) 20, (c, d) 30, (e, f) 40, and (g, h) 50. Scale bar = 500 nm.

**
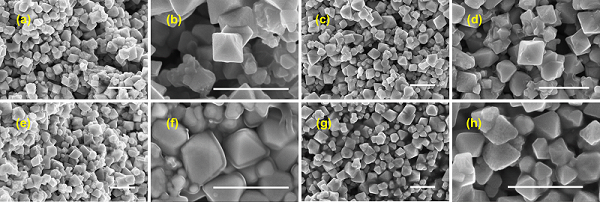
**

**Figure S7.** Low magnification FE-SEM images of Fe_3_O_4_@Ag-X, where X = (a, b) 20, (c, d) 30, (e, f) 40, and (g, h) 50. Scale bar = 500 nm.


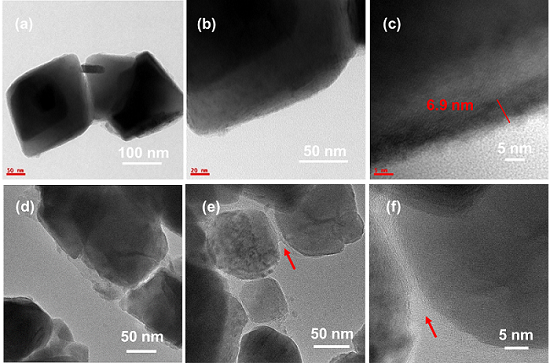


**Figure S8.** HR-TEM images of Fe_3_O_4_@Au-10 (a-c) and Fe_3_O_4_@Ag-10 (d-f) at different magnifications

**Figure S9.** XRD patterns of the three different alloy-based core-shell nanostructures.

**Figure S10.** ED-XRF spectra of the three different alloy-based core-shell nanostructures.


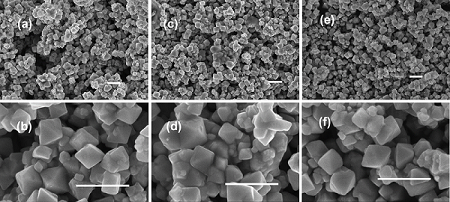


**Figure S11.** Low magnification FE-SEM images of (a, b) Fe_3_O_4_@AuAg-15:5, (c, d) Fe_3_O_4_@AuAg-10:10 and (e, f) Fe_3_O_4_@AuAg-5:15 alloy core-shell nanostructures. Scale bar = 500 nm.


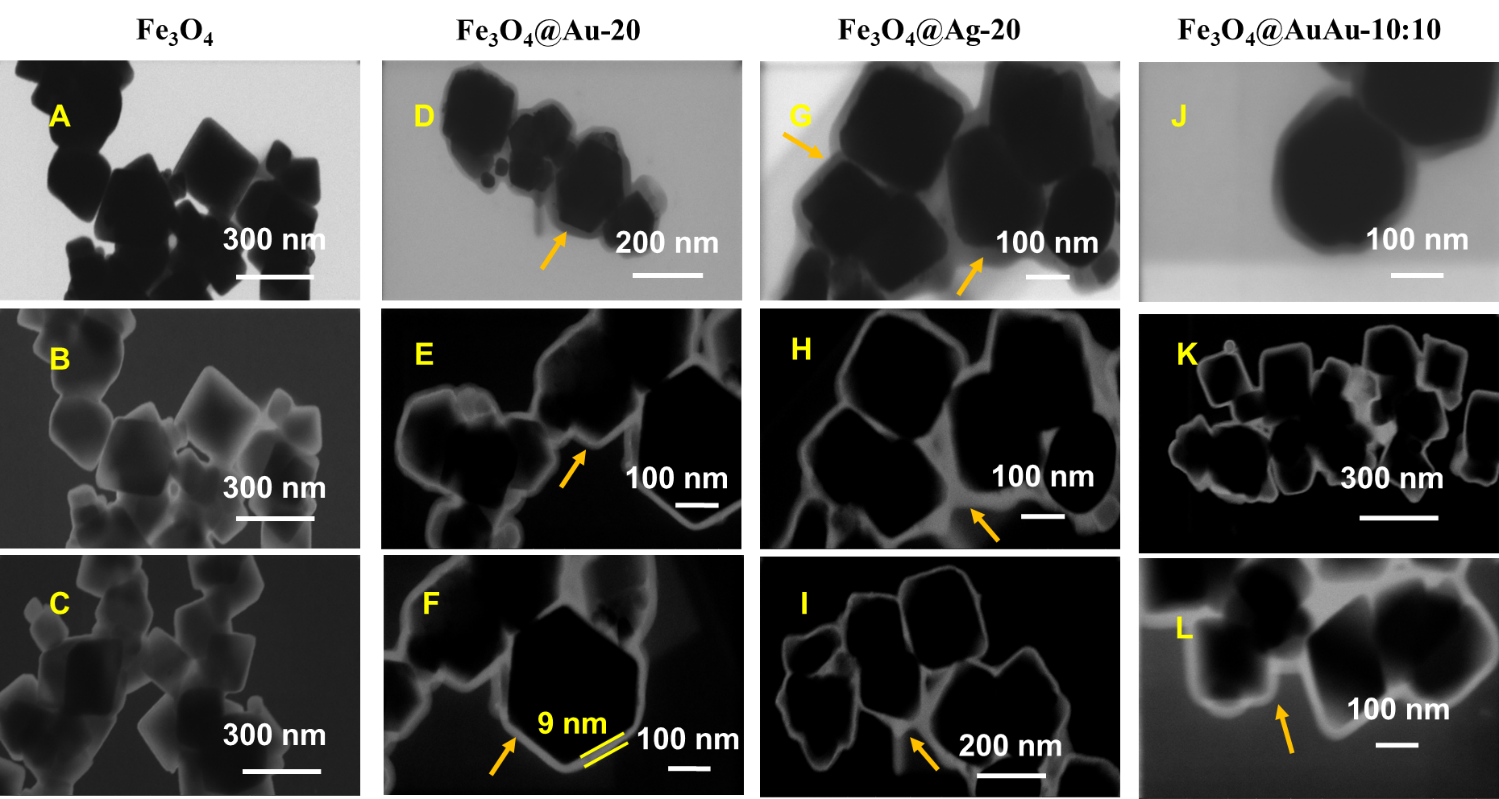


**Figure S12.** Composite STEM images of (A-C) Fe_3_O_4_, (D-F) Fe_3_O_4_@Au-20, (G-I) Fe_3_O_4_@Ag-20, and (J-L) Fe_3_O_4_@AuAg-10:10 recorded in dark and bright modes under different magnifications.


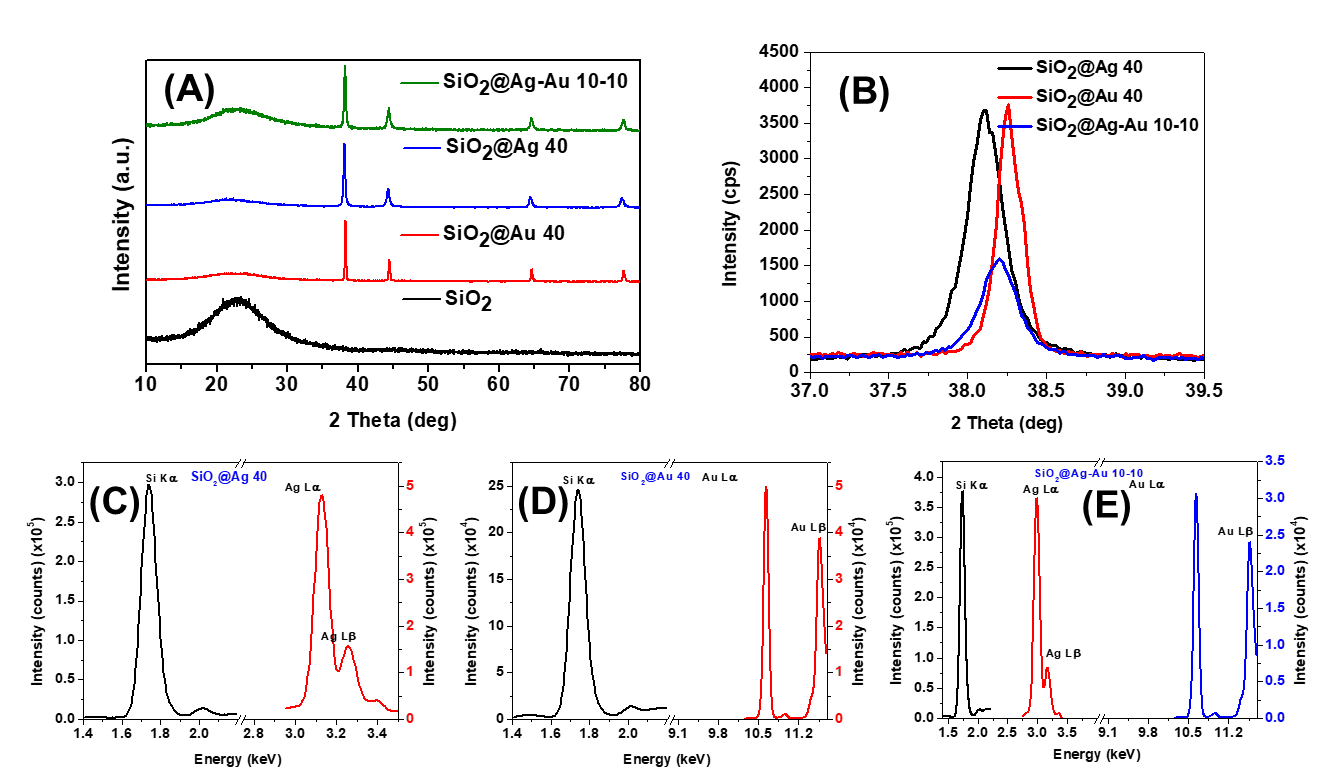


**Figure S13.** (A) The XRD patterns of SiO_2_@AgAu-10:10, SiO_2_@Ag-40, SiO_2_@Au-40 and pristine SiO_2_. (B) The magnified plot of (111) XRD planes for each catalyst to compare the variation of peak position with composition. (C), (D) and (E) ED-XRF spectra of SiO_2_@Ag-40, SiO_2_@Au-40, and SiO_2_@AgAu-10:10, respectively.

The XRD patterns of SiO_2_@M clearly revealed the presence of respective metals and alloys (after washing with aqueous ammonia). The magnified view of the (111) planes revealed a systematic shift in peak position with respect to the composition. The ED-XRF spectra confirmed the presence of metals with respect to the feed ratio.


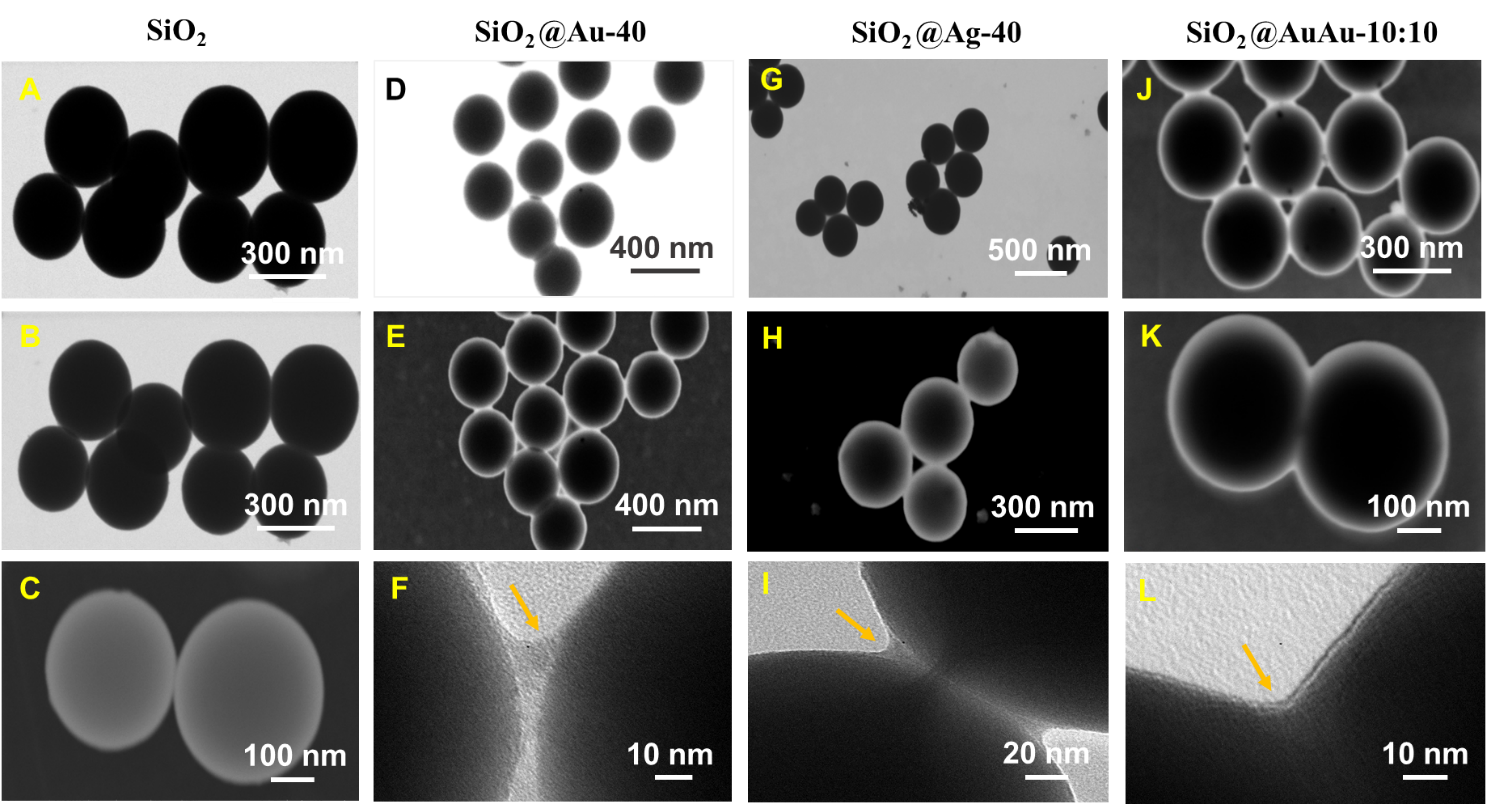


**Figure S14.** Composite STEM images of (A-C) SiO_2_, (D and E) SiO_2_@Au-40, (G and H) SiO_2_@Ag-40, and (J and K) SiO_2_@AgAu-10:10. TEM images of (F) SiO_2_@Au-40, (I) SiO_2_@Ag-40 and (L) SiO_2_@AuAg-10:10 with arrows indicating the shell layer present at the interface between two core SiO_2_ particles.

The STEM analysis (Apreo, FEI, 30 kV acceleration) in bright and dark field comparison has clearly revealed the presence of a thin metal shell layer. The metal-induced conjunction of the SiO_2_ particles has been found with metal and alloy shell layer. This has also been corroborated with surface area analysis, which revealed a decrease in surface area from 9.5 m^2^/g for the pristine SiO_2_ to 2.1, 2.5 and 4.7 m^2^/g for SiO_2_@Au-40, SiO_2_@Ag-40 and SiO_2_@AgAu-10:10, respectively.

**Figure S15.** SAXS analysis showing the higher scattering of Fe_3_O_4_@Au-10 than the corresponding Ag-shell counterpart (Fe_3_O_4_@Ag-10) at the same loading.

**Figure S16.** Hydrogen generation studies with Fe_3_O_4_@Au-20 and Fe_3_O_4_@Ag-20 from a 20 mL of a solution containing (a) 50 mM of AB and (b) 25 mM of NaBH_4_ as the hydrogen sources.

**(b)**

**(a)**

**(d)**

**(c)**

**Figure S17.** Kinetic studies with respect to (a, b) catalyst loading and (c, d) AB + NaBH_4_ mixture.

**
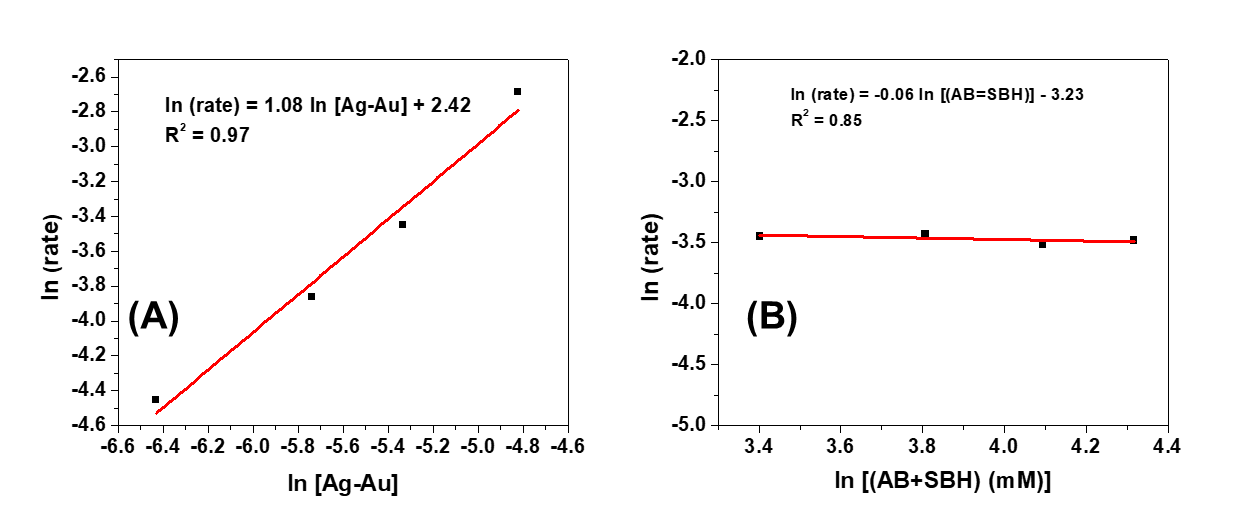
Figure S18.** Kinetic studies with respect to (A) catalyst loading and (B) AB + NaBH_4_ mixture using SiO_2_@AgAu-10:10.

The order was found to be first and zeroth with respect to the catalyst and the substrate, respectively. This has shown the reaction mechanism is identical irrespective of the nature of the core. It may be that the actual reaction rate with SiO_2_@AuAg was slower when compared to the Fe_3_O_4_@AuAg counterpart, which could be governed by several factors such as the electronic structure, size, and shape of the core.

**Figure S19.** Temperature-dependent hydrogen generation studies (a) to estimate the energy of activation from Arrhenius plot (b).

**(a)**

**(b)**

**Figure S20.** Control experiments for hydrogen generation from AB and SBH mixture using pristine Fe_3_O_4_, SiO_2_, and no-catalyst conditions.


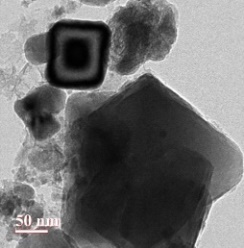

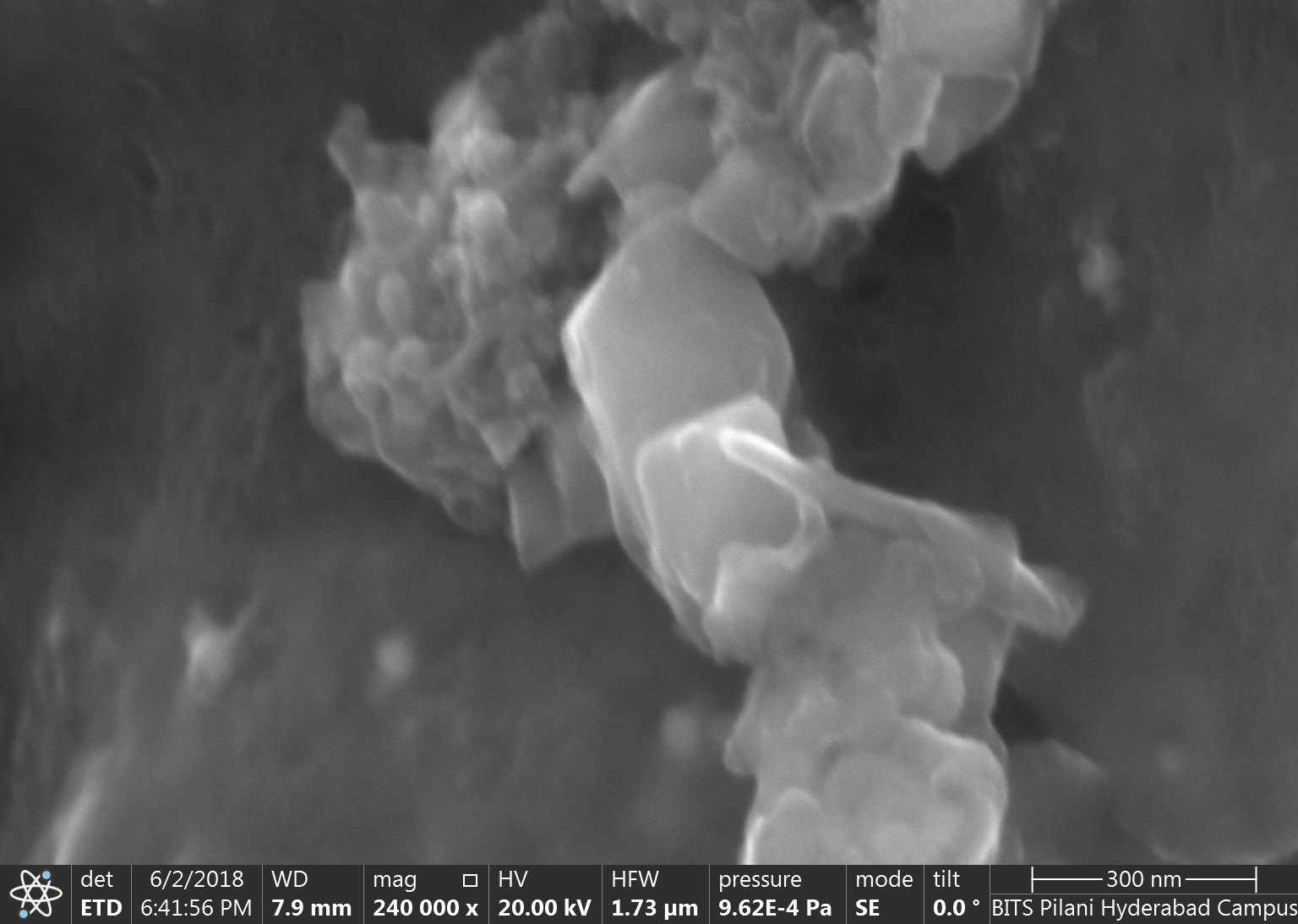

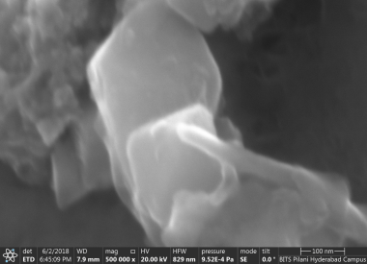


**100 nm**

**300 nm**

**(a)**

**(b)**

**(c)**

**(d)**

**Figure S21.** FE-SEM (a, b), HR-TEM (c) and ED-XRF (d) analyses on recycled Fe_3_O_4_@AuAg-10:10 alloy catalyst. The arrows indicate the polyborazylene regions.


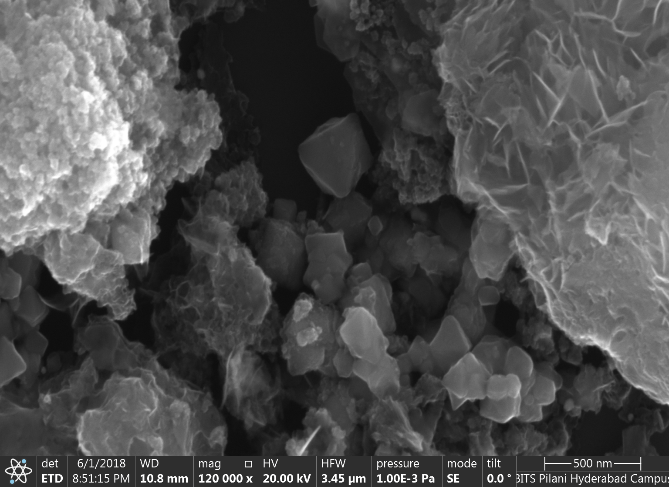

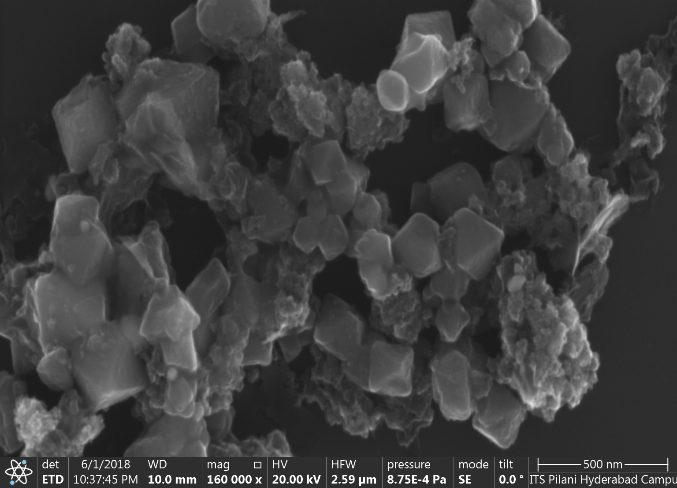


**(a)**

**(b)**

**Figure S22.** Low magnification FE-SEM images of Fe_3_O_4_@AuAg-10:10 alloy catalyst after 5 cycles. Scale bar = 500 nm.

**Figure S23.** Magnetization as a function of external applied magnetic field for three samples as indicated in the figure.
